# Left Renal Artery Thrombosis in a Patient With von Willebrand Disease Type 3 and Factor V Leiden Heterozygosity

**DOI:** 10.7759/cureus.94029

**Published:** 2025-10-07

**Authors:** Natalie L Albright, Sana Kamboj, Jason M Lucas

**Affiliations:** 1 Department of Medicine, Division of Hospital Medicine, Emory University School of Medicine, Atlanta, USA

**Keywords:** coagulopathy, factor v leiden, factor v leiden deficiency, factor v leiden heterozygosity, renal artery thrombosis, von willebrand disease, von willebrand disease type 3, von willebrand type 3

## Abstract

There are a few cases of combined thrombotic and hemophilic disorders coexisting in patients that have been published in the literature, such as factor V Leiden (FVL) with von Willebrand disease (VWD) type 1, and a report of a patient with VWD type 3, protein C and antithrombin III deficiency, and venous thromboses, but no reports in the literature were found of a patient with VWD type 3 and FVL and arterial thromboses. VWD type 3 is the most severe form because it results in nonexistent levels of von Willebrand factor (VWF) and is believed to be protective against arterial thromboses. We present a unique case of a 41-year-old man with a known history of VWD type 3 who presented with left renal artery thrombosis and a large left renal infarct, who was subsequently diagnosed with FVL heterozygosity. Both FVL and VWD type 3 can coexist in a patient. These patients require special considerations in treatment that have not been well documented throughout the literature.

## Introduction

Von Willebrand disease (VWD) is the most common congenital bleeding disorder, affecting approximately 1% of the US population [1]. VWD type 3 represents the most severe form, characterized by a near-total absence of von Willebrand factor (VWF). Patients with VWD type 3 typically exhibit significant bleeding tendencies and a hemophilia-like phenotype, with mucocutaneous bleeding and joint and muscle bleeds. VWD type 3 is typically managed with long-term VWF concentrate replacement therapy to prevent recurrent spontaneous bleeding in those with a history of severe and frequent bleeding episodes [2]. Conversely, factor V Leiden (FVL) mutation is the most common inherited thrombophilia, affecting approximately 5% of the general population in its heterozygous form [3]. It predisposes individuals to venous thromboembolism (VTE). The coexistence of VWD and FVL is rare, estimated to occur in only 0.25% of Caucasians [4]. This dual pathology presents a unique clinical challenge, as the opposing risks of bleeding and thrombosis complicate both diagnosis and management. Here, we describe a case of known VWD with newly presenting FVL, presenting with a renal artery thrombosis, a rare presentation of co-coagulopathy, and hemorrhagic disorders.

## Case presentation

A 41-year-old man with a known history of VWD type 3, hypertension, and alcohol use disorder presented to an outside hospital with left flank pain. He was diagnosed with VWD type 3 at age 7 after recurrent episodes of epistaxis and pain in his elbows, with a significant right thigh hematoma around 16 years of age. He was last on Humate P 10 years ago. The patient had not been on any therapies for VWD for many years and endorsed recent heavy alcohol use. His International Society on Thrombosis and Haemostasis (ISTH) Bleeding Assessment Tool (BAT) score was 13 points.

Initial imaging revealed a left upper renal pole infarct with patent vasculature on CT angiography (CTA). He was started on a regimen of morphine 6 mg every three hours (q3hr) as needed (prn), OxyContin 10 mg q4hr prn, Tylenol 1 g three times a day (TID), and Robaxin 750 mg q8hr for pain control, bowel management, and alcohol withdrawal. Given his history of VWD type 3, the hematology-oncology team was consulted, and anticoagulation was deferred. At this time, his measured prothrombin time (PT)/partial thromboplastin time (PTT) (12.4 {normal: 9.8-12.9 seconds} and 36.2 {normal: 25-38 seconds}, respectively), antithrombin III activity (98%; normal: 83%-128%), and lactate dehydrogenase (215 IU/L; normal: 135-225) levels were normal. The normal PTT was unexpected, given his VWD type 3, as this condition is typically associated with a prolonged PTT. A repeat PTT on hospital day 2 showed mild prolongation. On hospital day 4, a bolus of 8000 units of a heparin drip at 18 units/kg/hour was initiated and then continued as 100 units/mL in dextrose 5% in water (D5W). Testing for FVL mutation, prothrombin (PT) gene mutation, and protein C and S activity level was initiated.

**Figure 1:**
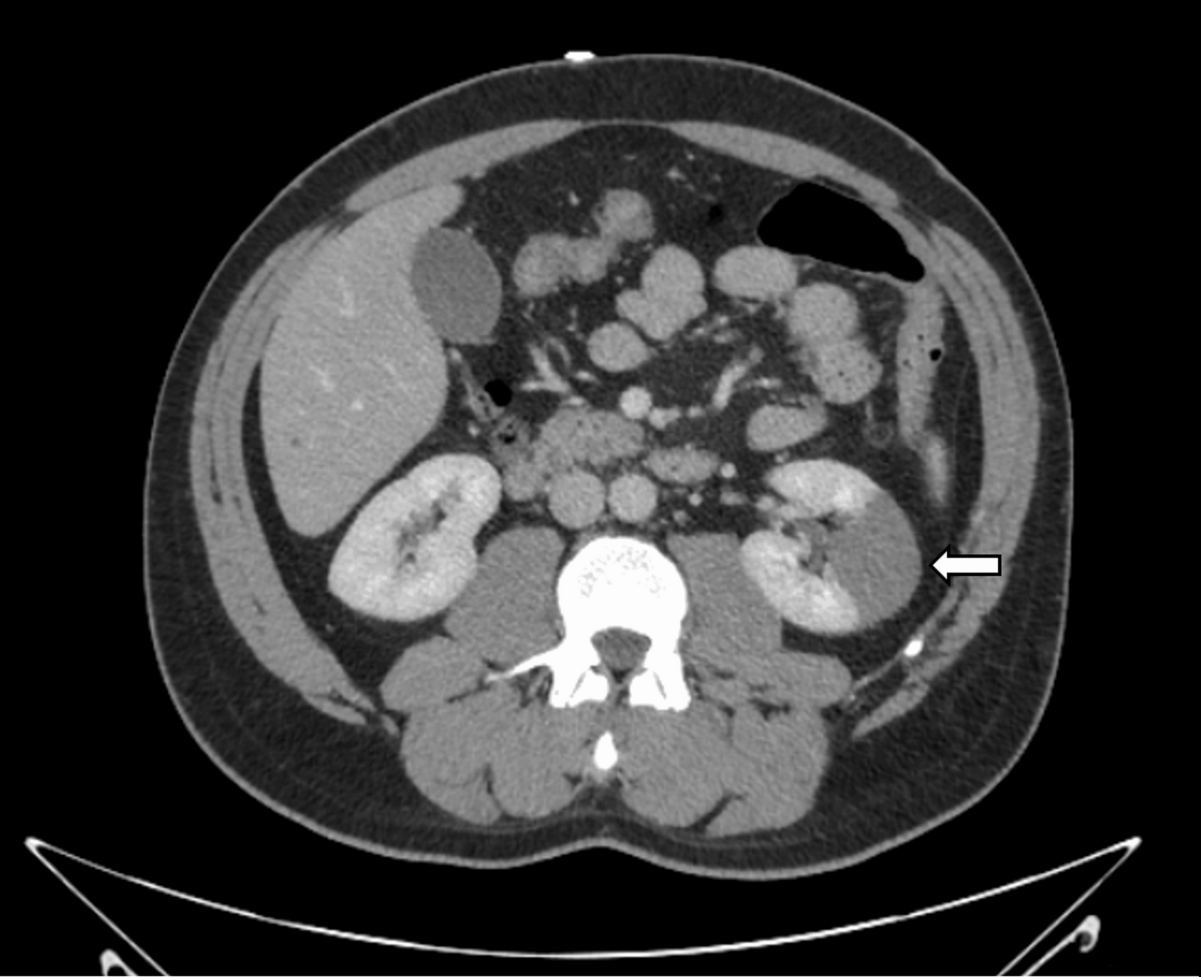
CTAP with a completely occluded segmental branch of the renal artery (white arrow) in the upper renal pole of left kidney. CTAP: computed tomography of the abdomen and pelvis

Despite initial management, the patient's flank pain worsened, and a repeat CTA of the abdomen and pelvis showed the occlusion of the left segmental renal artery with the progression of the left upper kidney pole infarction (Figure 1). At this time, he was transferred to our academic medical center for specialized hematologic care.

Upon arrival at our institution, vital signs were as follows: blood pressure (BP), 180/93; heart rate (HR), 98; respiratory rate (RR), 18; temperature, 37°C; and SpO_2_, 99% on room air (RA). On examination, the patient was uncomfortable and in clear pain and tachycardic with no murmurs and no notable skin abnormalities, and the abdomen was soft, non-tender, and non-distended.

The patient was noted to have leukocytosis (WBC, 15.9×10^3^/mcL; normal, 4.2-9.1×10^3^/mcL), hypertension (180/93 mmHg), and an elevated creatinine level (1.9 mg/dL; baseline, 1.1 mg/dL; normal, 0.70-1.30 mg/dL) from acute kidney injury. Baseline VWF antigen was 22%, high-molecular-weight VWF multimers were 14, factor VIII level was 39.9%, VWF activity was 8%, and PTT was 26.2 seconds (Table 1). The absence of the highest-molecular-weight multimers correlated with VWD type 3 activity. Genetic testing from the outside hospital showed a mutation consistent with FVL heterozygosity. Lupus anticoagulant and anticardiolipin antibodies were tested. Anticardiolipin antibody IgG was slightly elevated at 28.9 CU (normal: ≤20). CT of the chest, abdomen, and pelvis did not show evidence of malignancy.

**Table 1:** Key coagulation parameters on admission. VWF, von Willebrand factor; PT, prothrombin time; PTT, partial thromboplastin time; FVL, factor V Leiden

Test	Result	Reference Range
VWF Antigen	22%	53%-270%
VWF Activity	8%	49%-208%
Factor VIII Activity	39.9%	50%-185%
PT	12.4 seconds	9.8-12.9 seconds
PTT	36.2 seconds	25-38 seconds
Antithrombin III	98%	83%-128%
Lupus Anticoagulant	Negative	-
Anticardiolipin IgG	28.9 CU	≤20 CU
FVL Genetic Testing	Heterozygous	-

The patient was initially treated with von Willebrand Factor/Coagulation Factor VIII Complex (Human) 4,635 VWF:RCo units q12hr and a heparin drip at 18 units/kg/hour. This regimen was later transitioned to recombinant von Willebrand factor therapy 6,500 VWF:RCo units every 48 hours and apixaban 5 mg q12hr. He did not have any bleeding complications while in the hospital.

His hospital course was further complicated by pseudomonal bacteremia. A transthoracic echocardiogram (TTE) with bubble study was performed to rule out endocarditis, and no valvular vegetations were noted. The infectious disease team did not feel that it was necessary to proceed to TEE and decided to treat the renal artery thrombosis as septic emboli with a six-week course of levofloxacin 750 mg by mouth (PO) daily for seven days.

At discharge, he was placed on apixaban 5 mg twice daily for three months and recombinant von Willebrand factor 7,500 mg IV infusion every other day for ongoing management. Following discharge, he developed pneumonia and about 3-4 episodes of hemoptysis in a three-week period. Since then, he has been experiencing epistaxis about once per week but is overall able to continue working.

## Discussion

We present a case of a 41-year-old man with VWD type 3 who developed a renal artery thrombosis and was subsequently diagnosed with a FVL heterozygosity. This case is notable due to several rare and intersecting features. Firstly, VWD type 3 is the most severe form of the disease, characterized by absent or undetectable VWF activity, and is generally associated with bleeding complications. Second, FVL heterozygosity is typically associated with venous thromboembolism (VTE) rather than arterial events. Lastly, the occurrence of an arterial thrombus, specifically in the renal artery, in a patient with both conditions, is exceptionally rare and diagnostically complex.

While VWD types 1 and 2 have rarely been associated with thrombotic events, VWD type 3 is characterized by profoundly reduced or absent VWF and is typically associated with bleeding, not clotting. Overall, the prevalence of arterial thrombotic events in patients with VWD was reported to be 39%-63% lower than in the general population [5].​​ For VTE, elevated VWF levels increase the risk of VTE and long-term venous complications [6]. Patients with VWD type 3 are expected to have minimal thrombotic risk due to their profoundly reduced or absent VWF levels. Thus, the development of an arterial thrombus in a patient with VWD type 3 is highly unusual and should prompt a workup for coexisting prothrombotic conditions.

FVL, a common inherited thrombophilia, is often first diagnosed after a new thromboembolic event. It is primarily associated with venous thrombosis, particularly deep vein thrombosis and renal vein thrombosis [3]. Arterial events, such as myocardial infarction or stroke, are far less common and generally occur in the presence of additional risk factors [7]. Renal artery thrombosis is especially rare and has only been sporadically reported in association with FVL [8].

To our knowledge, this is the first reported case of VWD type 3 coexisting with FVL mutation and presenting as an arterial thrombosis. Previous case reports have described venous thrombotic events in patients with VWD types 1 or 2 and FVL but not type 3. For instance, one case describes a pregnant woman with VWD type 1 and FVL [9]. She had previously experienced easy bruising but was being anticoagulated with enoxaparin in her pregnancy. Another case details a family with both deficiency of FVL and VWF, showing another example of a clotting and bleeding disorder coexisting [10]. The thrombotic-hemophilic paradox remains poorly understood, and it is unclear whether such patients are more likely to exhibit bleeding or clotting phenotypes or remain asymptomatic. The management of these patients also remains controversial, as there are very few precedents of thromboprophylaxis in those without a history of thromboembolism [11].

In our case, the absence of prior thrombotic events likely contributed to the delayed identification of the patient's underlying FVL mutation. This case underscores the importance of recognizing that VWD, even in its most severe form, can coexist with undiagnosed prothrombotic conditions, placing patients at risk for thrombotic events. This case also highlights the clinical challenge of balancing hemostatic replacement with thromboprophylaxis. Our patient required repeated infusions of VWF concentrate at 4,635 units/kg every 48 hours in parallel with anticoagulation using high-intensity heparin infusions at 18 units/kg/hour q12hr. Careful titration, the close monitoring of factor levels, and multidisciplinary decision-making were essential to avoid both hemorrhagic and thrombotic complications.

Further research is needed to better understand the prevalence, clinical behavior, and optimal management of individuals with both bleeding and thrombophilic disorders. A better understanding of this overlap could guide more personalized treatment strategies.

## Conclusions

This case highlights a rare but clinically significant intersection of severe bleeding and thrombophilic disorders. In patients with VWD type 3 who develop arterial thrombosis, evaluation for inherited thrombophilias such as the FVL mutation should be considered. The coexistence of VWD type 3 and FVL and the development of renal artery thrombosis demonstrate the difficulty of balancing hemostatic replacement with anticoagulation. Successful management requires individualized treatment strategies, including the careful monitoring of anticoagulant dosing, VWF concentrate replacement, and factor levels to minimize both bleeding and thrombotic risks.
